# Mucocutaneous disease: a child with extrapulmonary manifestation of mycoplasma infection

**DOI:** 10.31744/einstein_journal/2025RC1138

**Published:** 2025-05-29

**Authors:** Kelly Regina Pereira da Silva, Rafaela Sardini Covello Giaccio, Bárbara Martins de Aquino, Marina Abellan Van Moorsel, Fernanda Mazziero Pires, Carolina Maria Hélène, Selma Maria Furman Hélène

**Affiliations:** 1 Hospital Israelita Albert Einstein Faculdade Israelita de Ciências da Saúde Albert Einstein São Paulo SP Brazil Faculdade Israelita de Ciências da Saúde Albert Einstein, Hospital Israelita Albert Einstein, São Paulo, SP, Brazil.; 2 Hospital Municipal Dr. Moysés Deutsch São Paulo SP Brazil Hospital Municipal Dr. Moysés Deutsch, São Paulo, SP, Brazil.; 3 Centro Universitário Lusíada Santos SP Brazil Centro Universitário Lusíada, Santos, SP, Brazil.; 4 Hospital Israelita Albert Einstein São Paulo SP Brazil Hospital Israelita Albert Einstein, São Paulo, SP, Brazil.

**Keywords:** Mycoplasma, Mycoplasma infections, Mycoplasma pneumoniae, Mucositis, Exantema, Erythema multiforme, Diagnosis, differential, Child

## Abstract

Mycoplasma-induced rash and mucositis is a distinctive subset of reactive infectious mucocutaneous eruption identified in 2015 to differentiate it from other mucocutaneous disorders such as Stevens-Johnson syndrome, erythema multiforme major, and toxic epidermal necrolysis. Although its pathophysiology is not completely understood, Mycoplasma-induced rash and mucositis is characterized by polyclonal B cell proliferation, production of antibodies against *Mycoplasma pneumoniae*, subsequent immune complex deposition, and keratinocyte apoptosis. Clinical manifestations include scattered cutaneous lesions and severe mucositis, which predominantly affect children and young males. In this report, we present the case of a 4-year-old boy with characteristic Mycoplasma-induced rash and mucositis symptoms, including erythematous annular lesions, mucosal involvement, and positive serologies for *Mycoplasma pneumoniae* and herpes simplex. The diagnostic challenges, treatment modalities, and differential diagnosis of erythema multiforme major are discussed. Our case underscores the importance of recognizing Mycoplasma-induced rash and mucositis as a distinct entity, facilitating accurate diagnosis and tailoring management strategies to optimize patient outcomes.

## INTRODUCTION

Mycoplasma-induced rash and mucositis (MIRM) is a subcategory of reactive infectious mucocutaneous eruption (RIME) that was first identified in 2015 to differentiate it from Stevens-Johnson syndrome (SJS), erythema multiforme major (EMM), and toxic epidermal necrolysis (TEN).^(1)^ Shared histopathological characteristics included keratinocyte apoptosis and sparse perivascular dermal infiltrates.^(2)^

Although the pathophysiology of MIRM has not been fully elucidated, it is thought to involve the proliferation of polyclonal B cells and production of antibodies against *Mycoplasma pneumoniae*. This leads to the formation and deposition of immune complexes that activate the complement system, thereby causing inflammation and tissue damage to the skin and mucosa. Molecular mimicry between *M. pneumoniae* P1-adhesion molecules and keratinocytes has also been hypothesized to contribute to keratinocyte apoptosis via cytotoxic T-cells.^(3)^ Clinically, MIRM presents with scattered cutaneous lesions on the extremities, trunk, and face, along with severe mucositis in the eyes, nostrils, mouth, and genitals.^(4)^

The cutaneous rash in MIRM is predominantly vesiculobullous (77%), although typical target lesions with three zones of demarcation and atypical target lesions with two color zones are also seen in approximately 48% of cases. Other less common presentations included papules (14%), macules (12%), and morbilliform lesions (9%).^(4)^ Prodromal symptoms, such as cough, chills, and fever, often precede mucocutaneous eruptions by approximately a week.^(1,4)^ MIRM is rare in adults, primarily affecting children and young males, with an average age of 12 years.^(1,3)^

## CASE REPORT

**Figure 1:**
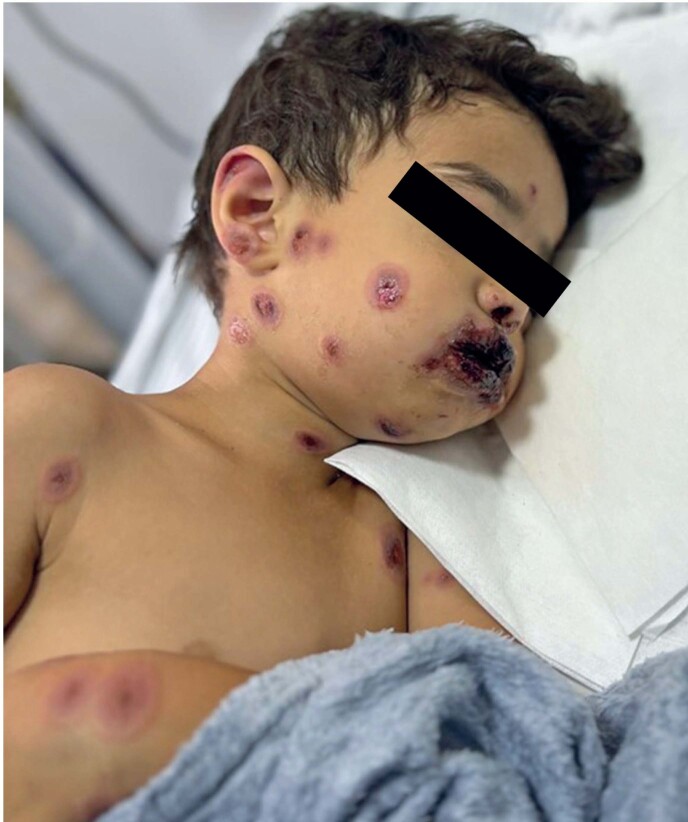
Erythematous annular lesions with a targetoid aspect and vesicobullous center on the arms and face

A 4-year-old previously healthy male, accompanied by his mother, presented with disseminated lesions on the body and oral and genital mucosa, primarily affecting the acral areas with some sparse lesions on the trunk. These symptoms were associated with a 5-day history of fever that worsened after amoxicillin administration. Upon admission, the patient was in a fair general condition, with scattered erythematous annular lesions, some showing a vesiculobullous aspect, and atypical target lesions with two-color zones, along with edema and crusted hemorrhagic lesions on the lips and nostrils. Edema and erythema were observed on the eyelids, along with ocular hyperemia and discharge (Figure 1).

Initially, SJS was considered; however, it was noted that the medication was administered after the onset of oral lesions. Two diagnostic hypotheses have been proposed: EMM and MIRM, with the latter being more likely due to the distribution and morphology of the lesions. Serological tests for *Mycoplasma pneumoniae* (IgG and IgM) and herpes simplex (IgM) were positive. However, considering the scarcity of lesions in the centripetal areas and the lesion morphology, the patient fulfilled the four diagnostic criteria for MIRM, confirming the diagnosis.

Supportive measures included intravenous hydration due to poor oral intake and topical treatment of the mucosal lesions with solid Vaseline. Intravenous antibiotic therapy with clarithromycin (15mg/kg/day for 10 days) was initiated because the patient could not receive oral doxycycline. Additionally, systemic corticosteroid therapy with methylprednisolone (1mg/kg/day) was administered for the first three days following diagnosis.

Laser therapy with an LED lamp was performed on the lips to optimize recovery. Systemic antihistamines were administered to alleviate itching, and an ophthalmic ointment containing ciprofloxacin and dexamethasone was administered daily for 10 days following evaluation by an ophthalmologist. The patient showed clinical improvement and was discharged 12 days later.

This study was approved by the Research Ethics Committee of *Hospital e Maternidade Santa Joana* (CAAE: 83912424.9.0000.5443; # 7.306.549).

## DISCUSSION

Diagnosis of RIME, particularly MIRM, can be challenging. Approximately 25% of patients with *M. pneumoniae* infections develop cutaneous and mucosal eruptions, emphasizing the importance of recognizing this condition.^(5)^ The typical mucosal lesions in MIRM include crusted hemorrhagic lesions on the lips, ulcerations on the tongue and oral mucosa,^(6)^ bilateral purulent conjunctivitis, photophobia, and eyelid edema.^(7)^ Lesions may also appear on the genital mucosa (vulva, vagina, penis, urethral meatus, and scrotum) as well as the nasal and anal mucosa.^(1,4,5)^ The pediatric-specific diagnostic criteria proposed by Ramien et al. provide useful guidance for the diagnosis of severe cutaneous adverse reactions.^(4)^

Differentiating MIRM from EMM is particularly difficult because of the potential overlap in infectious etiologies, particularly those of herpes simplex virus (HSV) and *M. pneumoniae*. However, in EMM, targetoid lesions are rarely associated with involvement of more than one mucosal site. Furthermore, EMM typically starts with acral eruptions (palms and soles) and spreads centrifugally, whereas MIRM tends to involve multiple mucosa, with localized skin lesions affecting the upper and lower limbs and face, sparing the palms and soles.^(8)^

**Figure 2:**
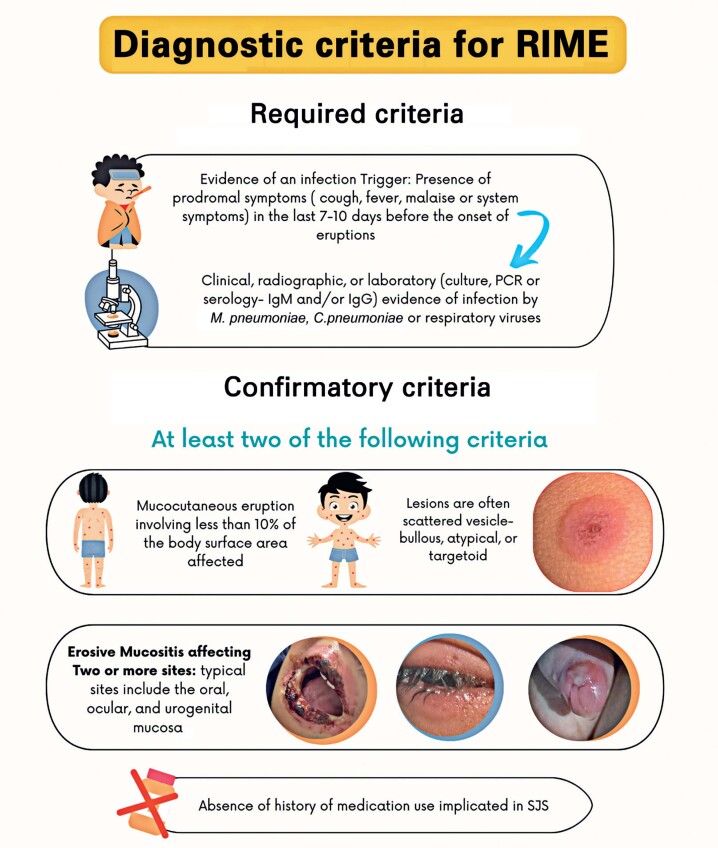
Diagnostic criteria for reactive infectious mucocutaneous eruption. This figure outlines the required and confirmatory criteria for diagnosing reactive infectious mucocutaneous eruption, specifically Mycoplasma-induced rash and mucositis

Source: Canavan TN, Mathes EF, Frieden I, Shinkai K. Mycoplasma pneumoniae induced rash and mucositis as a syndrome distinct from Stevens-Johnson syndrome and erythema multiforme: a systematic review. J Am Acad Dermatol. 2015;72(2):239-45. Review.

In this case, despite positive serological results for both HSV and *M. pneumoniae*, MIRM caused by *M. pneumoniae* was the most appropriate diagnosis. Positive IgM for HSV was interpreted as a false positive or a marker of a prior infection unrelated to the current mucocutaneous manifestations. This conclusion was based on the involvement of multiple mucosal sites (eyes, lips, oral cavity, tongue, nostrils, and prepuce) and the distribution of cutaneous lesions on the upper limbs, lower limbs, and face without involvement of the palms or soles (Figure 2). The patient's rapid clinical improvement following antibiotic therapy further supported the diagnosis of MIRM rather than an HSV-related condition.

Retrospectively, the patient's improvement following targeted treatment for *M. pneumoniae*, along with the fulfillment of four out of five diagnostic criteria, confirmed the diagnosis of MIRM. However, certain diagnostic tools such as PCR testing for HSV and biopsy of the lesions have not been used because of the limitations of this public healthcare setting.

## CONCLUSION

In conclusion, a thorough understanding of this distinct clinical entity will improve diagnostic accuracy and significantly affect patient prognosis. Recognizing Mycoplasma-induced rash and mucositis is essential, particularly for differentiating it from other mucocutaneous conditions, and special attention must be paid to the risk of secondary infections in lesions.
